# A Novel Defined Hypoxia-Related Gene Signature for Prognostic Prediction of Patients With Ewing Sarcoma

**DOI:** 10.3389/fgene.2022.908113

**Published:** 2022-06-02

**Authors:** Runyi Jiang, Jinbo Hu, Hongfei Zhou, Haifeng Wei, Shaohui He, Jianru Xiao

**Affiliations:** ^1^ Spinal Tumor Center, Department of Orthopaedic Oncology, No.905 Hospital of PLA Navy, Changzheng Hospital, Naval Medical University (Second Military Medical University), Shanghai, China; ^2^ The Third Convalescent Department, Hangzhou Sanatorium, Hangzhou, China

**Keywords:** ewing sarcoma, gene signature, hypoxia, biomarkers, prognosis

## Abstract

The therapeutic strategy of Ewing sarcoma (EWS) remains largely unchanged over the past few decades. Hypoxia is reported to have an impact on tumor cell progression and is regarded as a novel potential therapeutic target in tumor treatment. This study aimed at developing a prognostic gene signature based on hypoxia-related genes (HRGs). EWS patients from GSE17674 in the GEO database were analyzed as a training cohort, and differently expressed HRGs between tumor and normal samples were identified. The univariate Cox regression, Least Absolute Shrinkage and Selection Operator (LASSO) and multivariate Cox regression analyses were used in this study. A total of 57 EWS patients from the International Cancer Genome Consortium (ICGC) database were set as the validation cohort. A total of 506 differently expressed HRGs between tumor and normal tissues were identified, among which 52 were associated with the prognoses of EWS patients. Based on 52 HRGs, EWS patients were divided into two molecular subgroups with different survival statuses. In addition, a prognostic signature based on 4 HRGs (WSB1, RXYLT1, GLCE and RORA) was constructed, dividing EWS patients into low- and high-risk groups. The 2-, 3- and 5-years area under the receiver operator characteristic curve of this signature was 0.913, 0.97 and 0.985, respectively. It was found that the survival rates of patients in the high-risk group were significantly lower than those in the low-risk group (*p* < 0.001). The risk level based on the risk score could serve as an independent clinical factor for predicting the survival probabilities of EWS patients. Additionally, antigen-presenting cell (APC) related pathways and T cell co-inhibition were differently activated in two risk groups, which may result in different prognoses. CTLA4 may be an effective immune checkpoint inhibitor to treat EWS patients. All results were verified in the validation cohort. This study constructed 4-HRGs as a novel prognostic marker for predicting survival in EWS patients.

## Introduction

Ewing sarcoma (EWS) is a group of poorly differentiated small round cell tumors, and 85–90% of them embrace the classic t (11; 22) EWS/FLI1 translocation (Rodríguez-Galindo et al., 2008; Arshi et al., 2017). As the second most common primary bone tumor, EWS usually occurs among children and adolescents with an incidence of 1 per 1.5 million population, and most commonly involves the pelvis and proximal long bones (GrüNewald et al., 2018). Due to the improvement of multidisciplinary treatments, the 5-years overall survival (OS) rate of EWS patients increased from 44% in the 1970s to 68% in the 1990s for localized disease, and from 16 to 39% for metastatic disease (Esiashvili et al., 2008). Most current studies predicted the prognosis of EWS mainly based on the clinical information (Wan et al., 2017; Zhou et al., 2019; Jiang et al., 2021b), and few used gene models, although gene models have been used to evaluate the prognosis and provide novel therapeutic targets in many other cancer types. Therefore, identification of novel biomarkers and customization of therapeutic strategies are urgently demanded to improve the prognosis of EWS patients.

Hypoxia is a common micro-environmental feature in most solid tumors owing to the imbalance between the rate of tumor cell proliferation and vascular nutrient supply (Hanahan and Weinberg, 2011). Previous studies have demonstrated that hypoxia plays a key role in tumor cell proliferation, differentiation and apoptosis, tumor angiogenesis, and even drug resistance (Wouters and Koritzinsky, 2008; Wilson and Hay, 2011). In many tumors, prognostic signatures based on hypoxia-related genes (HRGs) were developed to serve as independent prognostic factors and provided new strategies for cancer treatment (Yang et al., 2017; Zhang et al., 2020; Abou Khouzam et al., 2021; Sun et al., 2021). Interestingly, some studies concerning the relationship between hypoxia and EWS demonstrated that hypoxia could stimulate the transcriptional signature of EWS-FLI1 and enhance the malignant properties of EWS(Aryee et al., 2010; Knowles et al., 2010), suggesting that targeted therapy focusing on HRGs may be a novel potential method for the treatment of EWS.

In this study, we systematically analyzed the characteristics of HRGs in EWS by utilizing high-throughput sequencing data and bioinformatics analyses. We not only firstly constructed an HRG-based prognosis model for EWS patients but found that HRGs could be used to distinguish EWS patients based on clinical and molecular features.

## Materials and Methods

### Data Collection

The transcriptome profiling (RNA-seq) data and corresponding clinical information obtained from GSE17674 in the GEO database were used as the training cohort, which contained 44 tumor tissues and 18 normal tissues (https://www.ncbi.nlm.nih.gov/geo/query/acc.cgi?acc=GSE17674). In addition, a total of 57 EWS patients with RNA sequencing results downloaded from the International Cancer Genome Consortium (ICGC; https://icgc.org) were used as the validation cohort. As the data from GSE17674 were microarray chip data and the data from the ICGC database were mRNA sequencing data, the normalization methods in the two cohorts were different. We used “normalizeBetweenArrays” in R package “limma” to deal with the data in training cohort, while fragments per kilobase of transcript per million fragments mapped (FPKM) were used in validation cohort. The characteristics of patients in two cohorts were shown in Table 1.

**TABLE 1:** Clinical characteristics of patients in the training and validation cohort.

Clinical factors	Training cohort (n = 44)	Clinical factors	Validation cohort (n = 57)
Age (years old, n, %)	—	Age (years old, n, %)	—
≤24	28 (63.7%)	≤24	45 (78.9%)
>24	16 (36.3%)	>24	12 (21.1%)
Sex (n, %)	—	Sex (n, %)	—
Male	2/8 (63.7%)	Male	31 (54.4%)
Female	16 (36.3%)	Female	26 (45.6%)
Tumor status (n, %)	—	Tumor status (n, %)	—
Localized	32 (72.7%)	Non-metastatic	38 (66.7%)
Recurrence or Metastasis	12 (27.3%)	Metastatic	18 (31.6%)
—	—	NA	1 (1.7%)

### Identification of Differentially Expressed HRGs

The HRG set was collected from the Molecular Signatures Database V7.4 (https://www.gsea-msigdb.org/gsea/msigdb/genesets.jsp) by searching the keyword “hypoxia”. Using the “limma” R package, differentially expressed HRGs between tumor and normal tissues were identified, with the cutoff criterion set to |Log_2_FC| ≥2 and use of an adjusted *p* value of <0.05.

### Tumor Classification Based on Differentially Expressed HRGs

The association between the differentially expressed HRGs and OS was analyzed by univariate Cox regression using the R package “survival,” and genes with *p* value <0.01 were used for tumor classification using the non-negative matrix factorization (NMF) method.

### Construction and Validation of the HRG Prognostic Model

Next, HRGs for tumor classification were included in the Least Absolute Shrinkage and Selection Operator (LASSO) regression, and the results were subjected to multivariate Cox regression analysis to establish a prognostic signature. Receiver-operator characteristic (ROC) curves were used to assess the performance of the prognostic signature by areas under the ROC curves (AUC). The risk score was calculated for each EWS patient based on the formula: Risk score = CoefHRGs1 × ExpHRGs1 + CoefHRGs2 × ExpHRGs2 + …CoefHRGs(n) × ExpHRGs(n). According to the optimal cutoff value of the risk score, EWS patients in the training cohort were divided into a low-risk group and a high-risk group. The Kaplan-Meier (KM) survival curve by the log-rank test was applied for comparing the prognosis between the two groups. A risk curve, survival state-related scatterplot and heatmap of HRGs were plotted after reordering individuals based on the risk scores. Using the same formula, the stability and reliability of this signature in the validation cohort were verified by similar methods using the “glmnet,” “survival,” “survminer,” “survivalROC” and “pheatmap” R packages.

### Independent Prognostic Analysis of the Risk Group

After constructing the risk model, we extracted the clinical information of patients in the training cohort, including age, sex and tumor status. Then, these variables were analyzed in combination with the risk level based on the risk model by univariate and multivariable Cox regression models. In addition, decision curve analysis (DCA) was used to test the independent prognostic value of risk levels. The same analyses were also performed in the validation cohort.

### Functional Enrichment Analysis Between High- and Low-Risk Groups

To compare the different gene expression profiles between high- and low-risk groups, we set the specific criteria (|Log_2_FC| ≥1 and adjusted *p* value <0.05) to select DEGs and perform Gene Ontology (GO) analysis on them to determine the biological processes (BPs), molecular functions (MFs), and cellular components (CCs) related to the HRG signature by using the R package “clusterProfiler”. The same procedure was performed in the validation cohort.

### Assessment of Immune Cell Infiltration Analysis

Hypoxia was highly associated with tumor immunity and immune environment. We used CIBERSORT to perform a correlation analysis between risk groups and infiltration abundances of 22 immune cells (Newman et al., 2015), and evaluated the activity of 13 immune-related pathways in high- and low-risk groups by employing single-sample gene set enrichment analysis (ssGSEA) in “GSVA” R package. In addition, immune checkpoints analysis was also employed to explore potential therapeutic targets for EWS patients in the two groups. Immune cell infiltration was also tested in the validation cohort.

### Statistical Analysis

All statistical analyses were performed by using R version 4.0.1 or SPSS (Version 25.0). The student’s *t*-test was used to compare gene expression between tumor and normal tissues. Wilcoxon test was used to compare the ssGSEA scores of immune-related pathways and immune checkpoints between the high- and low-risk groups. For each statistical analysis, two-tailed *p* < 0.05 was regarded as statistically significant.

## Results

### Algorithm to Classify EWS by Using Differentially-Expressed HRGs

**FIGURE 1:**
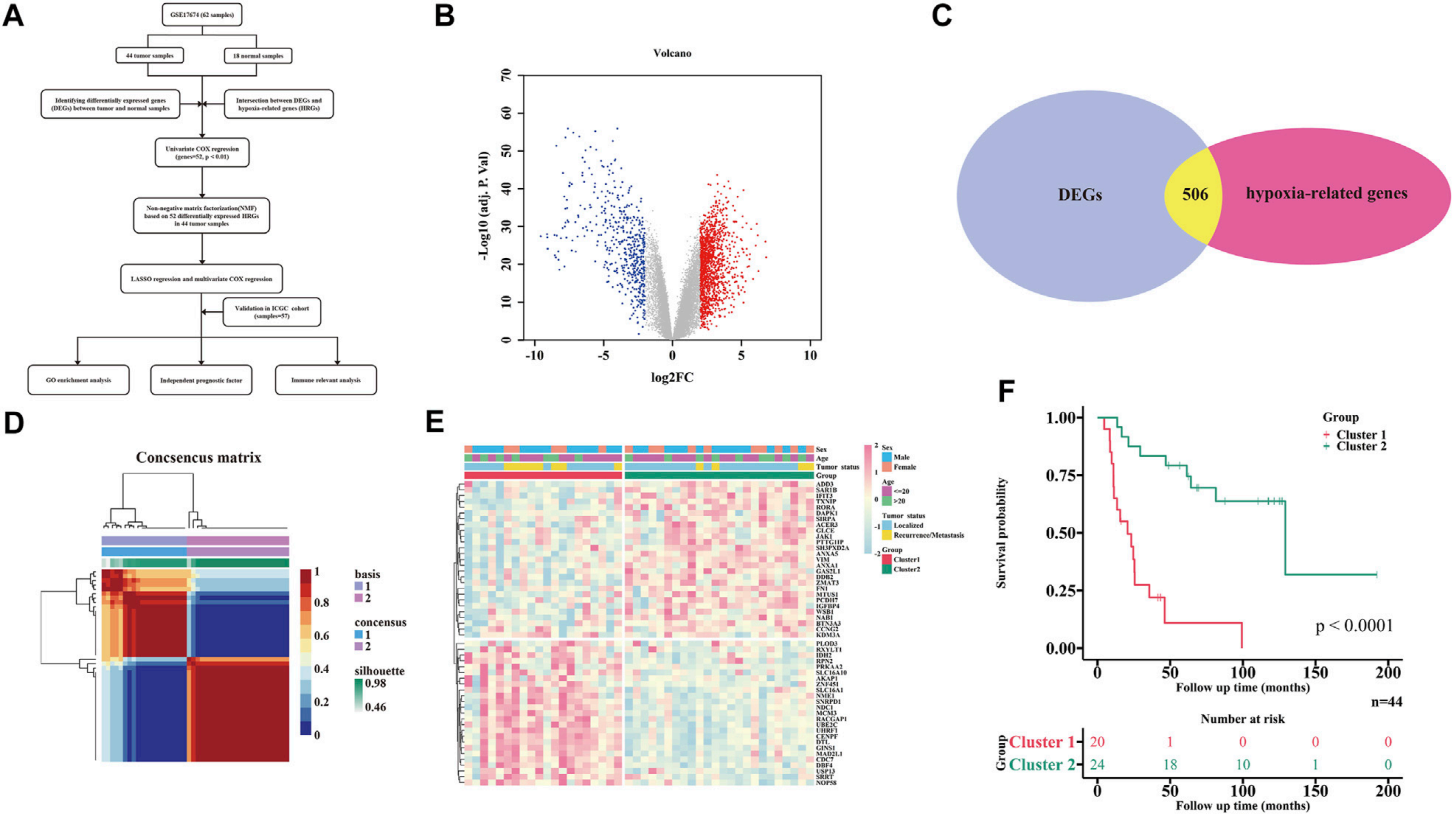
Tumor classification based on the differently expressed HRGs. **(A)** Flow chart of data analysis. **(B)** Volcano plot of DEGs between tumor and normal tissues. **(C)** Veen plot of DEGs and HRGs set. **(D)** All EWS patients were divided into two clusters by NMF cluster based on 52 HRGs (k = 2). **(E)** Heatmap shows the association of risk and clinicopathologic characters in two clusters. **(F)** KM curves of the two clusters.

The flowchart of this study is shown in Figure 1A. A total of 1829 DEGs, including 1,328 up-regulated genes and 501 down-regulated genes (Supplementary Table S1), were identified between 44 tumor and 18 normal tissues (Figure 1B), which contained 506 differentially expressed HRGs (Figure 1C). Univariate Cox regression analysis of the above 506 differentially expressed HRGs showed that 52 HRGs were significantly associated with the prognosis of EWS (*p* < 0.01, Supplementary Figure S1A).

To further explore the relationship between the expression of the 52 HRGs and the EWS subtype, NMF analysis was performed in the 44 tumor tissue samples. Using the NMF method, the potential features of gene expression profiling were identified by decomposing the original matrix into two non-negative matrices, and the optimal k value was determined by comprehensive correlation coefficient analysis (Brunet et al., 2004). By increasing the clustering variable (k) from 2 to 6 (Supplementary Figure S1B,C), we found that when k = 2, a clear and sharp boundary was shown in the consensus matrix heat map, indicating that the 44 tumor samples could be divided into two clusters by adopting this cutoff (Figure 1D). The gene expression profiles of the 52 HRGs between the two clusters are illustrated in a heatmap by merging the clinical features (age, sex and tumor status) in Figure 1E. In addition, a very significant difference in prognosis was observed between the two clusters (*p* < 0.0001) (Figure 1F).

### Establishment of a Prognostic Signature in the Training Cohort

**FIGURE 2:**
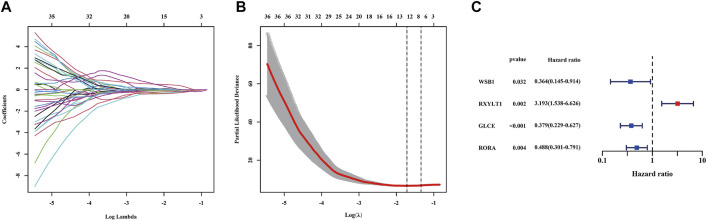
Identification of protective or risk HRGs by LASSO and multivariate Cox regression analysis in the training cohort. **(A)** LASSO regression of the 52 HRGs. **(B)** Cross-validation for tuning parameter selection in the LASSO regression. **(C)** Candidate HRGs to construct gene signature by multivariate Cox regression analysis. LASSO = least absolute shrinkage and selection operator.

**FIGURE 3:**
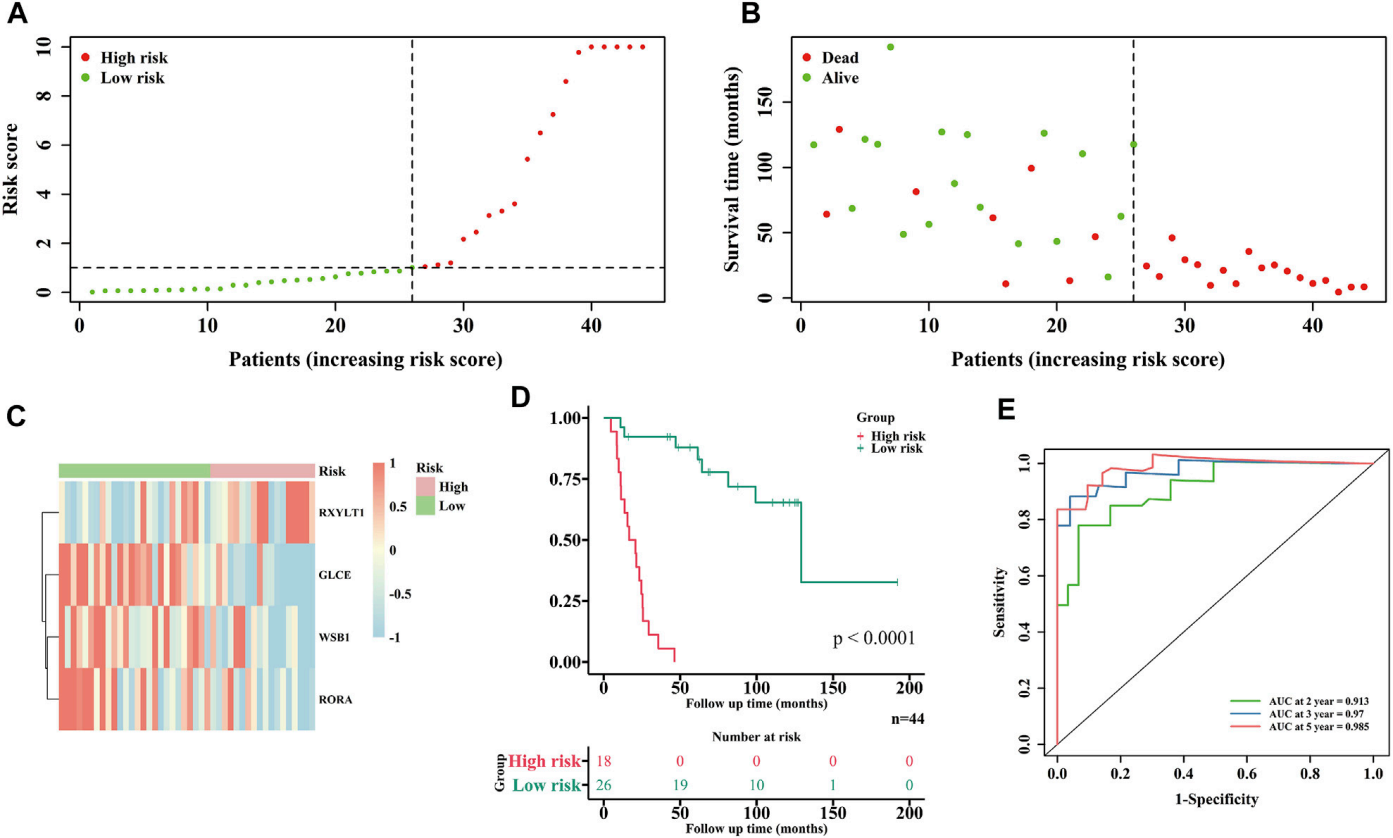
Construction of prognostic signature in the GEO cohort. **(A)** Distribution of each EWS patient according to the risk score. **(B)** Survival state-related scatterplot of EWS patients. **(C)** Heatmap of 4 HRGs expressions in two risk groups. **(D)** KM curves of low- and high-risk groups. **(E)** Evaluation of the prognostic signature by ROC curves.

According to the optimal *λ* value of LASSO regression (Figures 2A,B), eight (DDB2, WSB1, RXYLT1, TXNIP, IFIT3, GLCE, NAB1 and RORA) of the 52 HRGs screened above were subjected into multivariate Cox regression analysis to develop an HRG prognostic signature, and the results are shown in Figure 2C, including 3 protective HRGs (WSB1, GLCE and RORA) and 1 risk HRG (RXYLT1). The risk score formula is as follows: Risk score = -1.011*WSB1 expression +1.160 * RXYLT1 expression - 0.9707* GLCE expression - 0.7172* RORA expression. Patients in the training cohort were stratified into a low-risk group (n = 26) and a high-risk group (n = 18) based on the optimal cutoff value of risk score. It was found that patients with high-risk scores were associated with a higher probability of death than those with low-risk scores (Figures 3A,B), and the enrichment status of 4 HRGs in the two subgroups is shown in Figure 3C. The KM curve indicated that patients in the high-risk group had a significantly lower OS rate than those in the low-risk group (*p* < 0.0001) (Figure 3D). In addition, the AUC of 2-, 3- and 5-years ROC curves of this signature were 0.913, 0.97 and 0.985, respectively (Figure 3E). The KM curves of four HRGs to construct the signature are shown in Supplementary Figure S2.

### Validation of the Prognostic Signature in the ICGC Database

**FIGURE 4:**
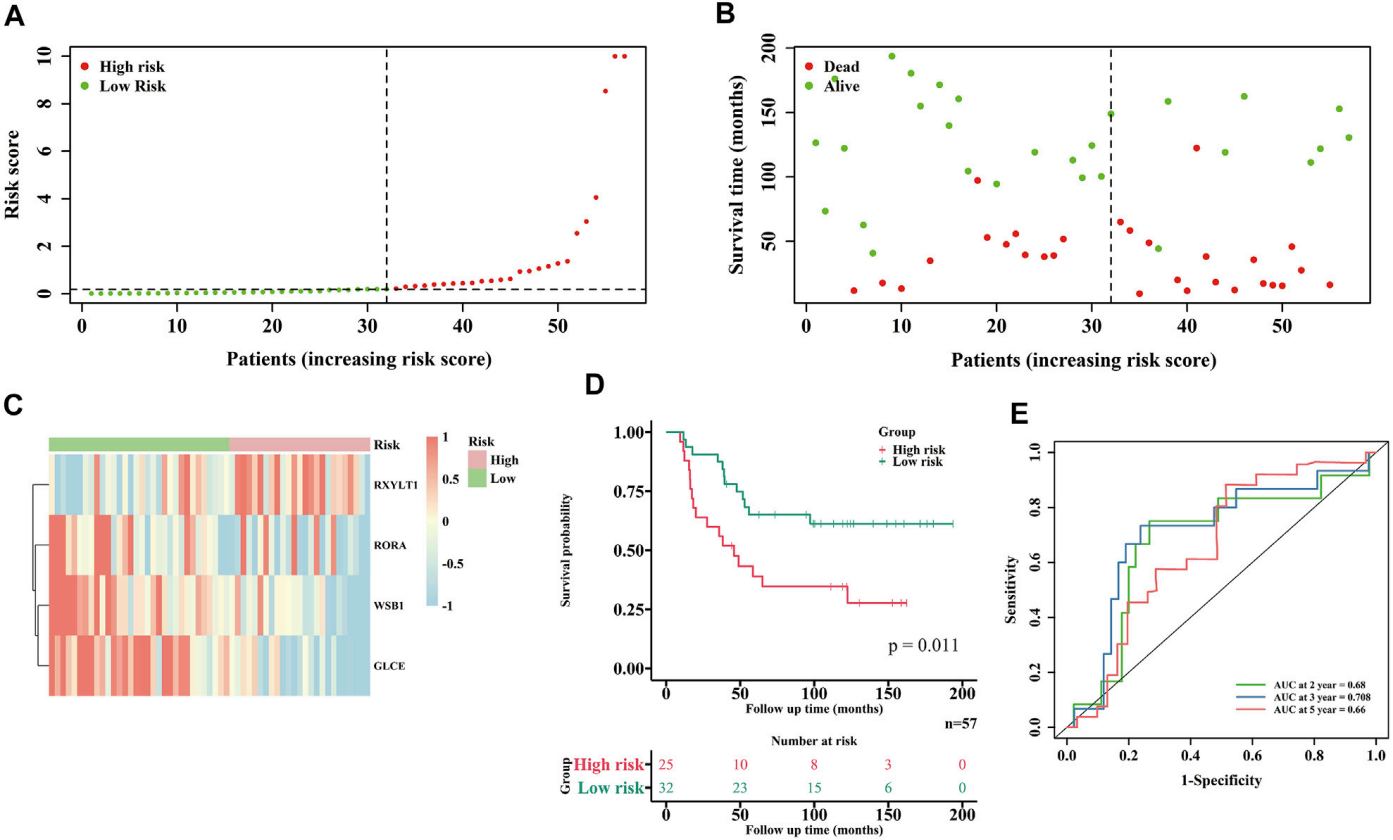
Validation of the risk model in the ICGC cohort. **(A)** Distribution of EWS patients in ICGC cohort based on the risk score formula. **(B)** Survival status of each EWS patient. **(C)** Heatmap of 4 HRGs expressions in two risk groups. **(D)** KM curves for comparison of the overall survival in two risk groups. **(E)** ROC curves showed the predictive efficiency of the risk model.

The robustness of this HRG prognostic signature was assessed in EWS samples from the ICGC database. A total of 57 samples were enrolled in the external validation cohort. According to the algorithm developed in the training cohort, 57 EWS patients were also separated into a low-risk and a high-risk group. Figures 4A–D depict the different survival statuses and differentially expressed HRGs in the two groups, showing the same tendency as the training cohort, suggesting that patients in the high-risk group had poorer prognoses. The 2-, 3- and 5-years ROC curves of the prognostic signature in the validation cohort are shown in Figure 4E, AUC being 0.68, 0.708 and 0.66, respectively. All these results indicate that our predictive model was reliable.

### The Independent Prognostic Value of the Risk Score

Univariate and multivariate Cox regression analyses were further performed among the variables available to determine whether the risk score (as a factor together with clinical features) was an independent prognostic predictor for EWS. The results of multivariate Cox regression analysis showed that only the risk level was significantly associated with the prognosis of EWS patients in both training and validation cohorts (HR = 44.120, 95% CI = 8.677–224.341, *p* < 0.001; HR = 2.217, 95% CI = 1.040–4.726, *p* = 0.039, respectively) (Tables 2, 3). The DCA also demonstrated that the risk level was a good independent prognostic factor for EWS patients in both training and validation cohorts (Supplementary Figure S3).

**TABLE 2:** Univariate and multivariate cox regression analysis of clinical information for EWS patients in the training cohort.

Factors	*n*	Univariate cox regression analysis		Multivariate cox regression analysis	
		HR (95% CI)	*p* value	HR (95% CI)	*p* value
Age: ≤20/ > 20 years	28/16	1.197 (0.537–2.671)	0.660	1.585 (0.674–3.728)	0.291
Sex: Male/Female	28/16	1.046 (0.472–2.317)	0.912	1.076 (0.452–2.565)	0.868
Tumor status: Localized/Recurrence or Metastasis	32/12	1.415 (0.639–3.133)	0.392	0.660 (0.263–1.659)	0.377
Risk level: low risk/high risk	26/18	33.791 (7.379–154.748)	<0.001	44.120 (8.677–224.341)	<0.001

Yrs, years old; CI, confidence interval.

**TABLE 3:** Univariate and multivariate cox regression analysis of clinical information for EWS patients in the validation cohort.

Factors	*N*	Univariate cox regression analysis		Multivariate cox regression analysis	
		HR (95% CI)	*p* value	HR (95% CI)	*p* value
Age: ≤20/ > 20 years	45/12	0.327 (0.099–1.087)	0.068	0.284 (0.067–1.216)	0.090
Sex: Male/Female	31/26	1.039 (0.500–2.160)	0.919	0.955 (0.448–2.036)	0.905
Tumor status: Non-metastatic/Metastatic/NA	38/18/1	1.752 (0.805–3.697)	0.161	1.459 (0.640–3.326)	0.369
Risk level: low risk/high risk	32/25	2.525 (1.202–5.301)	0.014	2.217 (1.040–4.726)	0.039

Yrs, years old; CI, confidence interval.

### GO Enrichment Analysis of the Training and Validation Cohorts

**FIGURE 5:**
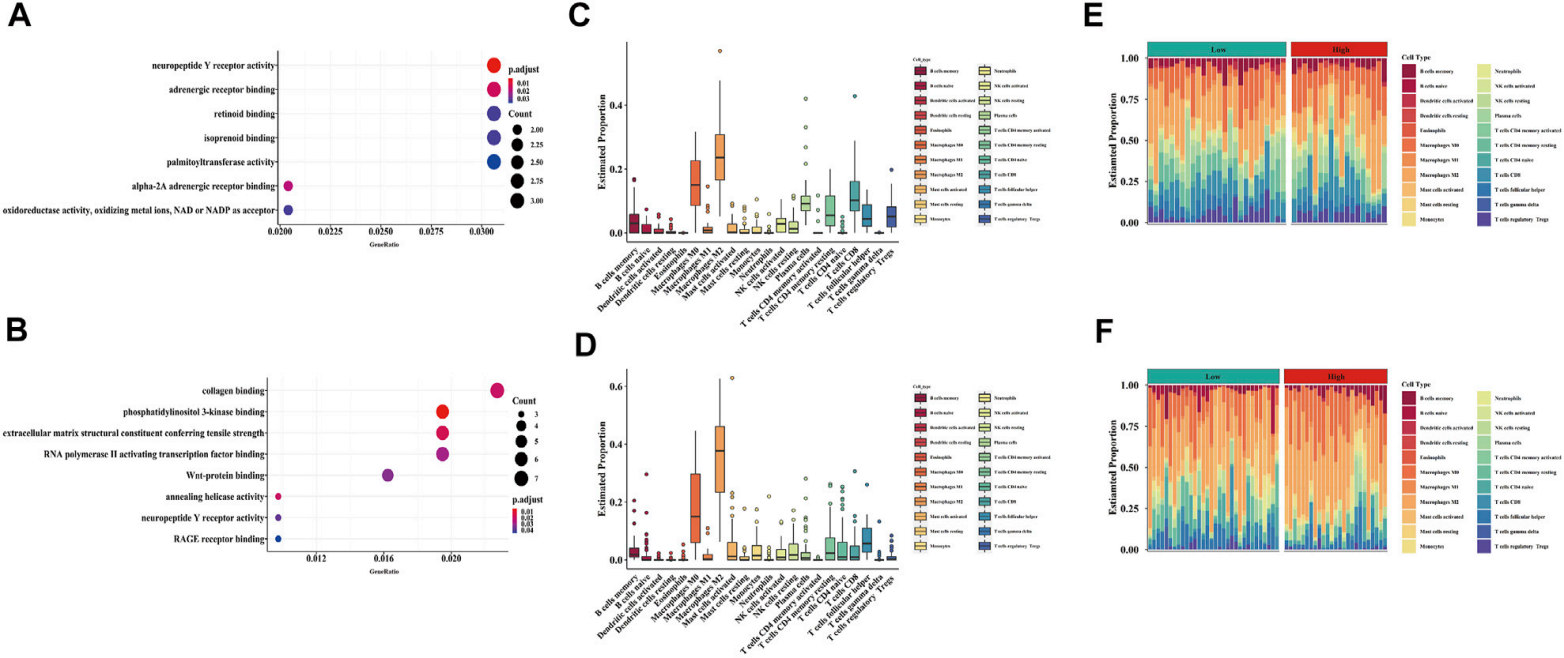
Functional analysis and immune cell component between two risk groups in both GEO and ICGC cohort. **(A,C,E)** GO enrichment analysis and immune cells proportion in the GEO cohort. **(B,D,F)** GO enrichment analysis and immune cells proportion in the ICGC cohort.

In the training cohort, a total of 119 DEGs were identified between the high- and low-risk groups (Supplementary Table S2), and most MFs were related to hypoxia, including neuropeptide Y receptor activity and oxidoreductase activity, oxidizing metal ions, NAD or NADP as acceptor (Figure 5A). Similarly, a total of 362 DEGs were found between the high- and low-risk groups in the validation cohort (Supplementary Table S3), and MFs had a relationship with hypoxia, including phosphatidylinositol 3-kinase binding, neuropeptide Y receptor activity and RAGE receptor binding (Figure 5B).

### Relationship Between Hypoxia and Immune Cell Infiltration

**FIGURE 6:**
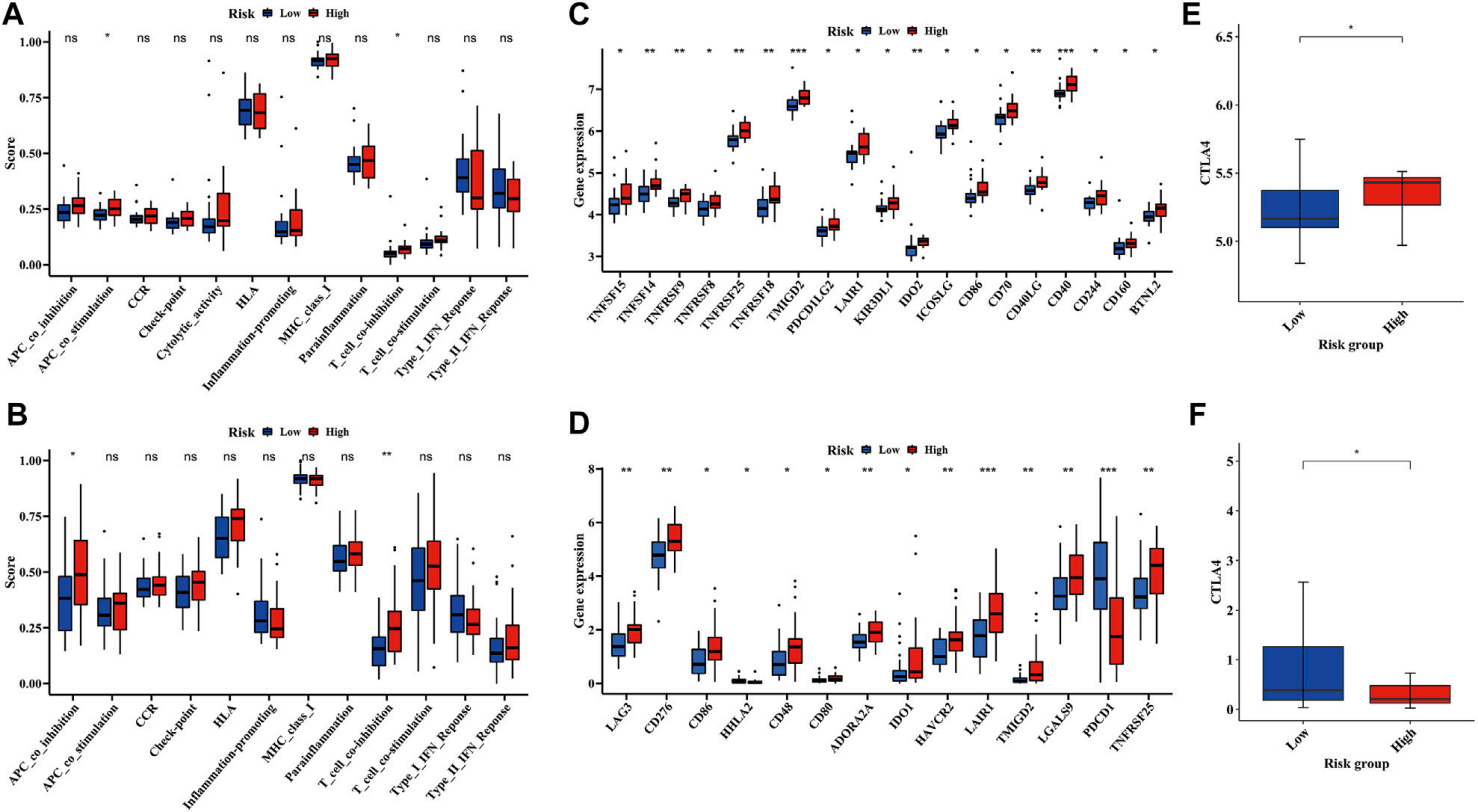
Difference of immune-related pathways and immune checkpoints between low- and high-risk groups in both GEO and ICGC cohort. **(A,C,E)** Comparison of immune-related pathways and immune checkpoints between two risk groups in the GEO cohort. **(B,D,F)** Comparison of immune-related pathways and immune checkpoints between two risk groups in the ICGC cohort.

To further understand the association between hypoxia and immune infiltration, we compared the enrichment scores of 22 types of immune cells, 13 types of immune-related pathways and immune checkpoints between the two subgroups in both the training and validation cohorts by employing CIBERSORT and ssGSEA. In the training cohort, macrophage was the major immune cell in both low- and high-risk groups (Figures 5C,E), which was also detected in the validation cohort (Figures 5D,F). Only two immune-related pathways, APC co-stimulation and T cell co-inhibition pathways, showed significance between the low- and high-risk groups in the training cohort (Figure 6A), but one of them (T cell co-inhibition pathway) also had significance between two risk groups in the validation cohort (Figure 6B). In addition, some immune checkpoint inhibitors including LAIR1, TNFRSF25 and CTLA4 were significantly identical between the training and validation cohorts (Figures 6C–F).

## Discussion

EWS is an aggressive bone and soft-tissue tumor, accounting for about 2% of cancers in children (Riggi et al., 2021). Currently, the main therapeutic strategy for EWS is the combination of cytotoxic chemotherapy with surgery and/or radiation. Although it has dramatically increased the OS rate of EWS patients, treatment-associated morbidities occur now and then (Goss and Gordon, 2016). Despite the advent of multiple therapeutic targets including EWS-FLI1, FOXO1, Gli and some immunotherapeutic targets (Guo et al., 2006; Yang et al., 2010; Sankar et al., 2014), none of them have been practically employed in clinical practice. Most cancer tissues suffer more hypoxia than normal tissues, and hypoxia has been demonstrated to play a key role in cancer progression and therapy resistance, compromising survival outcomes of distinct cancers (Wilson and Hay, 2011). Therefore, hypoxia is regarded as a novel potential therapeutic target by many oncologists.

It was found in our study that the differentially-expressed HRGs identified between tumor and normal tissues were associated with the prognosis for EWS patients. Intriguingly, almost all protective and risk HRGs were enriched in different groups, indicating different survival outcomes in the two subtypes. These findings suggest that different HRGs may contribute to a different survival status in EWS patients. To further evaluate the prognostic value of these HRGs, a four-gene prognostic model was developed *via* LASSO regression analysis and multivariate Cox analysis, comprising 3 protective HRGs (WSB1, GLCE and RORA) and 1 risk HRG (RXYLT1). To the best of our knowledge, this is the first gene signature based on HRGs to predict the prognoses of EWS patients.

WSB1 is a member of the WD-protein subfamily which encodes WD repeat and SOCS box-containing protein and joined ubiquitination and degradation of homeodomain-interacting protein kinase 2. Chen et al. (2006) reported that the high expression of WSB1 could help neuroblastoma patients gain a good prognosis, which is consistent with our findings in the EWS patients. Another protective gene GLCE was proposed to exert a protective effect on EWS and could function as a prognostic biomarker to predict prognosis (Jiang et al., 2021a; Zhou et al., 2021). Although RORA has not yet been found as a hallmarker of hypoxia in EWS, previous studies revealed that it could inhibit the proliferation and tumorigenesis of glioma and breast cancer cells (Du and Xu, 2012; Jiang et al., 2020). More experiments are required to confirm whether RORA participates in the process of tumor suppression of EWS. RXYLT1, also known as TMEM5, is a transmembrane protein that can induce skeletal muscle diseases (Astrea et al., 2016; Manya et al., 2016; Nishihara et al., 2018). Although there is no sufficient evidence to confirm the relationship between EWS and RXYLT1, we figure that RXYLT1 may affect the occurrence of EWS in that EWS is a bone and soft tissue tumor. Above all, although these genes could be used to construct a signature for prognostic prediction of EWS patients, further studies are required to gain more insights into the specific functions of these genes in EWS.

Based on the prognostic model, we stratified the EWS patients into a low- and high-risk group to discriminate the clinical outcomes. Protective HRGs were majorly expressed in the low-risk group, while risk HRGs were predominantly enriched in the high-risk group. The KM curve showed that EWS patients in the low-risk group lived a significantly longer time, and the ROC curves indicated the robustness and reliability of this prognostic signature. These results were validated in another mRNA sequencing dataset of EWS from the ICGC database. In addition, we demonstrated that the risk level based on the risk score was an independent prognostic factor in both cohorts. DCA curves also indicated the accuracy of the risk level as a powerful independent prognostic indicator. To sum up, we believe that the signature based on HRGs for EWS patients in this study could serve as a convincible prognostic algorithm in clinical practice.

Additionally, we analyzed the DEGs between the two different risk groups and found that the molecular function related to hypoxia was involved. Neuropeptide Y, which has high endogenous synthesis and release in EWS, was found to be highly associated with tumor biology as a sympathetic neurotransmitter with pleiotropic actions. Interestingly, hypoxia could induce upregulation of Y2R/Y5R expression to trigger the NPY/Y2R/Y5R axis, which stimulated tumor cell proliferation, survival, migration and angiogenesis in EWS (Tilan and Kitlinska, 2016). Previous studies reported that hypoxia could modulate VEGF induction in tumor cells by activating the stress inducible phosphatidylinositol 3-kinase pathway, thus facilitating the development of new blood vessels in solid tumors (Mazure et al., 1997). Advanced Glycation End Products (AGE) are the final products generated during glycation, and they often function by combining with their receptors (Khan et al., 2018). Activation of the AGE-RAGE axis plays a pivotal part in tumor growth and metastasis through the binding of RAGE with AGE, the process of which could be driven under the hypoxic condition (Ganapathy-Kanniappan and Geschwind, 2013; Yamagishi et al., 2015). The above evidence suggests that hypoxia could commonly occur in high-risk patients, resulting in worse survival outcomes. Hypoxia may also play a critical role in the progression and metastasis of EWS.

Although the mechanism of hypoxia has been explored in diverse cancer types in the past few decades, the potential modulation between tumor immunity and hypoxia remains elusive. The immune system is often inhibited in hypoxia as a major component of the tumor microenvironment (Wigerup et al., 2016). Tumor-associated antigens on EWS cells with low expression of human leukocyte antigen (HLA)-A, B,C, failed to be recognized by antigen presenting cells and effector T cells, and high expression of HLA- G actively suppressed tumor-specific T cells (Morales et al., 2020). HLA-G, as a non-classical major histocompatibility complex (MHC) class I molecule, often directly inhibited natural killer (NK) and tumor-specific T cells, and expressed in up to 34% EWS samples (Spurny et al., 2018). In this study, the functional score of HLA and MHC class I was the top 2 in the 13 immune-related pathways, even though there is little difference between the low- and high-risk groups. However, pathways related to HLA and MHC class I such as T cell co-inhibition were significantly different between the two risk groups, and much higher in the high-risk group, indicating that EWS tumor cell growth may be enhanced by undermining effective antitumor immune responses. In addition, immune checkpoint inhibitors such as PD-1 and PD-L1 have shown their clinical value in a variety of solid tumors, but showed no significant clinical activity in EWS (Tawbi et al., 2017). Our study found that the expression of CTLA-4 was different between the low- and high-risk groups. In EWS cells, CTLA-4 binding of CD80 on APCs led to T cell anergy by preventing CD80^−^ CD28 costimulation of T cells (Morales et al., 2020), which may be responsible for the stronger activity of T cell co-inhibition in the high-risk group. Studies reported that the polymorphism of CTLA-4 was a risk factor affecting the prognosis of EWS(Yang et al., 2012). We assume that CTLA-4 may be a useful immunotherapeutic target to assist EWS patients, and further *in vivo* and clinical trials on immune checkpoint inhibitors are required to explore the benefits in EWS.

As this is the first study to use HRGs to build up a prognostic gene model in EWS patients, limitations are unavoidable. Firstly, both constructed and validated prognostic signatures were based on the retrospective data from public database, and the cohort size was relatively small which was probably owing to the low incidence of EWS and a lack of studies. In addition, the specific mechanisms of WSB1, GLCE, RORA and RXYLT1 in EWS remain unclear, and more prospective studies should be designed to validate the results and conclusions of the present study.

## Conclusion

Hypoxia-related genes could be utilized to classify EWS patients based on different clinical and molecular features. In addition, we constructed and validated a novel signature of 4 HRGs, which proved to be independently associated with the OS of EWS patients. CTLA4 may be an effective therapeutic target in treating EWS. The underlying mechanisms between HRGs and tumor immunity in EWS warrant further investigation.

## Data Availability

The original contributions presented in the study are included in the article/Supplementary Material, further inquiries can be directed to the corresponding authors.
