# Supplementary material for: A Novel Defined Hypoxia-Related Gene Signature for Prognostic Prediction of Patients With Ewing Sarcoma
Source: Front Genet. 2022 Jun 2;13:908113. doi: 10.3389/fgene.2022.908113 (PMC9201760; doi:10.3389/fgene.2022.908113)
Supplement: Supplementary file 4 [file DataSheet1.docx]

**
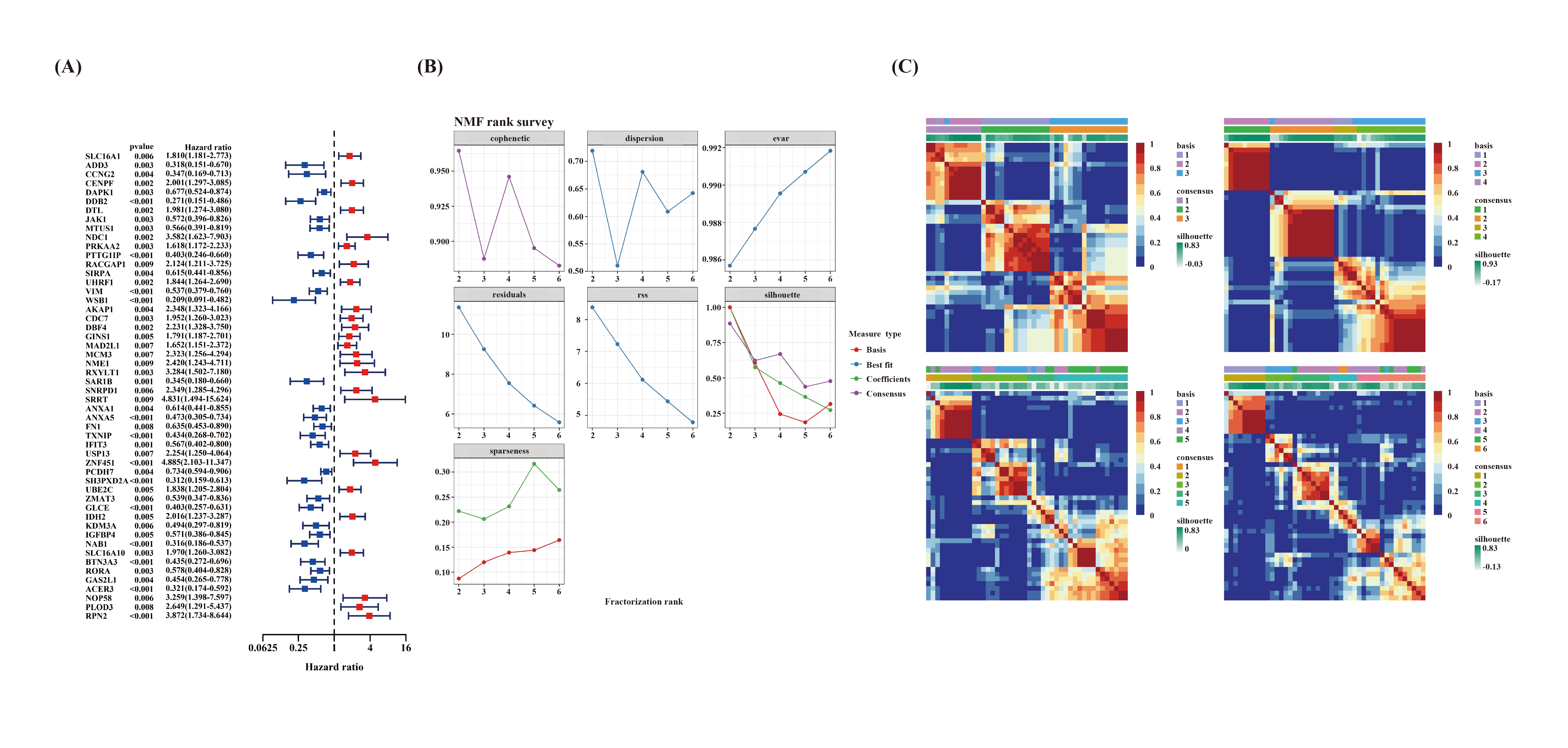
**

**Figure S1 The NMF results of 52 HRGs.** (A) The results of univariate Cox regression analysis of 52 HRGs (p < 0.01). (B) (C) The results of NMF clustering (k=3-6).

**
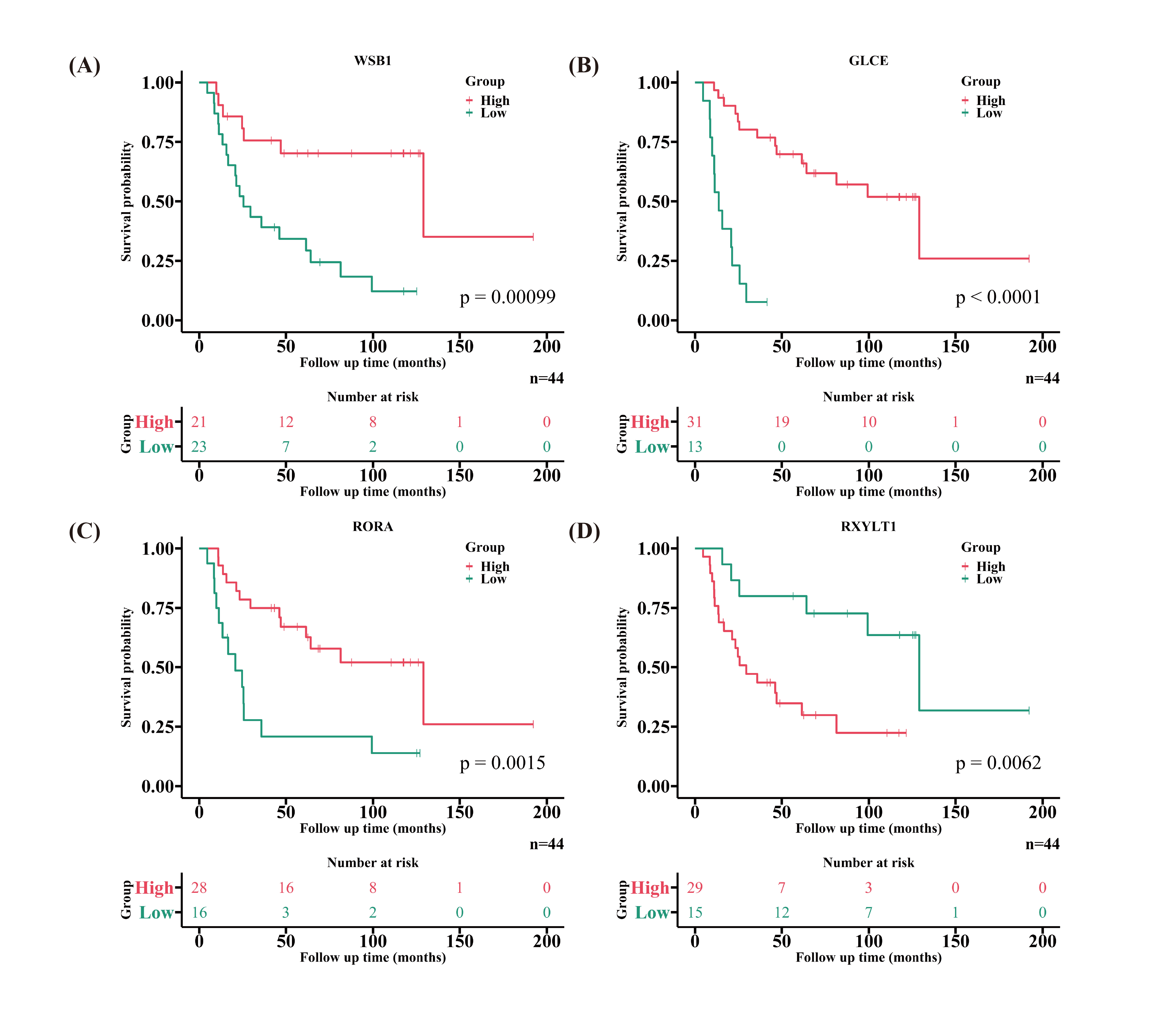
**

**Figure S2** **KM curves of 4 HRGs.** (A-D) KM curves of 4 HRGs used to develop prognostic signature.

**
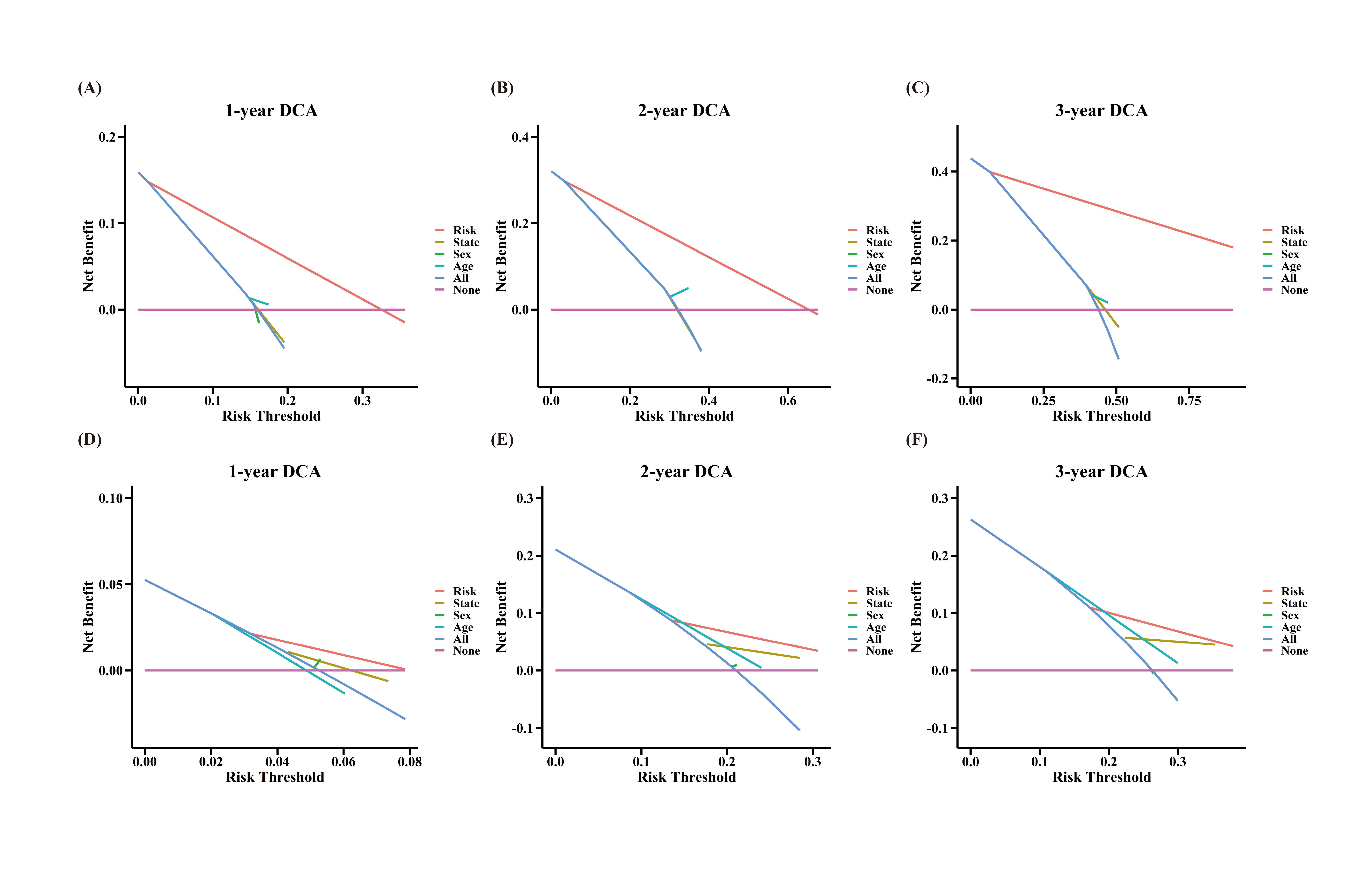
**

**Figure S3 DCA curves of clinical factors.** (A-C) 1-year, 2-year and 3-year DCA of each clinical factors (including risk levels) in GEO cohort. (D-F) 1-year, 2-year and 3-year DCA of each clinical factor (including risk levels) in ICGC cohort.

**Table S1** Differentially expressed genes (DEGs) between tumor and normal tissues.

**Table S2** Differentially expressed genes (DEGs) between low- and high-risk group in training cohort

**Table S3** Differentially expressed genes (DEGs) between low- and high-risk group in validation cohort
